# Characterization of mesenchymal stem cells derived from adipose tissue of a cougar (*Puma concolor*)

**DOI:** 10.21451/1984-3143-AR2019-0109

**Published:** 2020-05-05

**Authors:** Diana Maritza Echeverry, Pamela Alejandra Asenjo, Daniela Michele Rojas, Constanza Javiera Aguilera, Lleretny Rodríguez-Álvarez, Fidel Ovidio Castro

**Affiliations:** 1 Department of Animal Science, Faculty of Veterinary Sciences, Universidad de Concepción, Concepción, Chile

**Keywords:** cell therapy, feline, Puma concolor, stem cell, wild cat

## Abstract

Adipose derived mesenchymal stem cells (AMSCs) have been isolated from domestic and wild cats. For wild cats, the isolation of AMSCs has been reported in the black-footed cats (*Felis nigripes*) and guigna (*Leopardus guigna*). Stromal vascular fraction (SVF) isolated from cougar adipose tissue have been used to restore elbow functionality in the cougar (*Puma concolor*) but multipotent characteristics of these cells have not been described. The present study describes for the first time the isolation and characterization of mesenchymal stem cells derived from adipose tissue of cougar. AMSCs and fibroblasts from six months female cougar were isolated and cultured in DMEM/F12, supplemented with FBS 10% + 1% Antibiotic/Antifungal + 2.4 mM L-Glutamine + 2.4 mM pyruvate up to passage 5. Expression of pluripotent and surface marker genes was evaluated at mRNA level. Mesodermal differentiation (adipogenic, osteogenic and chondrogenic) was described. AMSCs expressed mRNA of pluripotent genes *Oct4, Nanog, Sox2* and *Klf4* and surface markers *Cd44*, *Cd90*, *Cd105* and *MHCII*. Fibroblasts showed similar mRNA expression with the exception of *Sox2*. AMSCs obtained from cougar exhibit multipotency features similar to domestic cats MSC, nevertheless, other analyses are required. AMSCs from cougar could be a source of interest for treatment of individuals that remain in captivity or arrive to wildlife rehabilitation centers.

## Introduction

The isolation of mesenchymal stem cells (MSCs) from several tissues represents an opportunity for their use in cell therapy in domestic and / or threatened animals. Although in domestic cats their isolation and characterization from different tissues has been widely described, for wild cats there are only few reports of isolation of this type of cells (Gómez et al., 2015; Echeverry et al., 2019). The collection of tissue samples from wild cats is difficult because mainly they are endangered species and specimens are rarely available to perform this procedure. Despite this, previous studies reported similar characteristics between AMSC of domestic and wild cats such as *Felis nigripes* and *Leopardus guigna* (Gómez et al., 2015; Echeverry et al., 2019). AMSCs from wild cats demonstrated multilineage differentiation capacity toward mesoderm cell lineages as adipogenic, osteogenic and chondrogenic, but also toward ectoderm cell lineage in case of *Felis nigripes* (Gómez et al., 2015; Echeverry et al., 2019). AMSCs from *Leopardus guigna* showed mRNA expression of *Oct4, Nanog, CD44 and CD90* (Echeverry et al., 2019). These previous studies suggest that AMSCs from wild cats have important features of multipotent cells.

Likewise, the use of MSCs for cell therapy in threatened and captive species has been scarce and poorly reported. The *Puma concolor* is one of the most common species in zoos and rehabilitation centers and displays pathologies similar to domestic cats, some of which could be potentially treated by means of cell therapy (Holsback et al., 2013; Miller et al., 2018). The objective of this study was to characterize a cellular population isolated from abdominal adipose tissue of a puma in order to determine its potential for cell therapy in this species.

## Materials and methods

### Collection and isolation of AMSCs

This study was approved by the Ethical Committee for Animal Experimentation of the Universidad de Concepción, permit number: CBE-03-2019. Peritoneal adipose tissue was isolated during the ovarian-hysterectomy procedure of a female cougar approximately 6 months old that was assigned to a rehabilitation center. The adipose tissue was homogenized and digested in type I collagenase at 0.01% at 37 ° C for 30 min. It was filtered and centrifuged at 140 × g for 5 minutes to isolate stromal vascular fraction (SVF). The pellet was resuspended in 20% SFB / DMEM + 1% Antibiotic/Antifungal + 2.4 mM L-Glutamine + 2.4 mM Pyruvate. The isolated cellular fraction was cultivated as primary cells. Cells were tested free of mycoplasmas contamination by multiplex PCR following the protocol previously reported (Table 1) (Uphoff and Drexler, 2002, 2004). Kinetics of cell growth was evaluated by quantitation of doubling population time after cell staining and counting using built-in software. No karyotype was performed. Cells were frozen after the first trypsinization (passage 1).

**Table 1:** Primer sequences used for Mycoplasma detection on fibroblasts and AMSC from cougar.

**Forward Primers**		**Reference**
Myco-5-1	CGCCTGAGTAGTACGTTCGC	Uphoff and Drexler (2002, 2004).
Myco-5-2	CGCCTGAGTAGTACGTACGC	
Myco-5-2	TGCCTGAGTAGTACATTCGC	
Myco-5-2	TGCCTGGGTAGTACATTCGC	
Myco-5-5	CGCCTGGGTAGTACATTCGC	
Myco-5-6	CGCCTGAGTAGTATGCTCGC	
**Reverse Primers**		
Myco-3-1	GCGGTGTGTACAAGACCCGA	Uphoff and Drexler (2002, 2004).
Myco-3-2	GCGGTGTGTACAAAACCCGA	
Myco-3-3	GCGGTGTGTACAAACCCCGA	

### In vitro multilineage differentiation

Adipogenic, osteogenic and chondrogenic differentiation assays were performed after thawing primary cells, in passage 2. For the adipogenic differentiation the cells were cultured in induction medium containing DMEM 10% SFB supplemented with 1 μM dexamethasone, 0.5 mM 3- isobutyl-L-methylxanthine and 0.1% insulin-transferrin-selenium-X. At day 7 of differentiation the cells were fixed in 4% paraformaldehyde and stained with Oil Red for 20 min. For osteogenic differentiation the cells were cultured in induction medium consisting of DMEM with 10% SFB, 1% antibiotic / antifungal, 0.1 μM dexamethasone, 0.2 mM ascorbic acid and 10mM β-glycerol phosphate. On day 21 the samples were fixed in 4% paraformaldehyde stained with 1% alizarin red to detect calcium deposits. Chondrogenic differentiation was performed as previously described with minor modifications (Castro et al., 2014). For chondrogenic induction cells were suspended in 500 µl chondrogenic medium, centrifuged at 140 × g for 5 min in 15-ml polypropylene conical tubes for culture in micro mass. Pelleted cells were incubated at 38.5°C under 5% CO_2_ in chondrogenic medium (DMEM/F12 supplemented with 10% FBS, 4.5 gr/L D-glucose, 10 µl/mL insulin– transferrin–selenium (ITS; Gibco), and 100 nM dexamethasone, 1 µM ascorbic acid 2-phosphate, and 2.5% equine platelet rich plasma (ePRP)) for 30 days. Cells were fixed and stained with Alcian Blue. Cells of control groups were cultured in DMEM/F12 supplemented with 10% FBS + 1% Antibiotic/Antifungal + 2.4 mM L-Glutamine + 2.4 mM Pyruvate, without other supplements, for the same time periods as in experimental groups. All stained cells were visualized with phase-contrast optics on an inverted microscope (Olympus CKX-41). All reagents were from Sigma -Aldrich, St Louis, MO, USA.

### RT-PCR analyses

The expression of *Oct4, Nanog, Klf4, Sox2, Cd44, Cd90, Cd105* and *MHCII* mRNA were tested by RT-PCR. The set of primers used has been tested before for *Felis catus* and *Leopardus guigna* species in our laboratory (Echeverry et al., 2019 and unpublished results). Feline *Sdha* (succinate-dehydrogenase-complex-flavoprotein subunit A) housekeeping was used as internal standard (Table 2). Total RNA was isolated from cougar AMSCs and dermal fibroblasts at P2 to compare expression. RNA was extracted from each sample using an EZNA RNA extraction kit (Omega, Georgia, USA). The first-strand, cDNA, was synthesized from 500 ng of DNase-treated total RNA using 50 ng random hexamers (Invitrogen, Waltham, Massachusetts, USA) and 200U of MMLV reverse transcriptase (New England Biolabs Ipswich, Massachusetts, USA) according to the manufacturer’s instructions. RT-PCR amplification was performed in a 10 μl reaction mixture in 40 cycles under the following conditions: 94 °C for 30 s, 58 °C for 30 s, and 72 °C for 40 s, with additional seven min incubation at 72 °C after cycle completion. Final PCR product was visualized in 1% agarose gel.

**Table 2:** Primer sequences used for the detection of gene expression in AMSC and fibroblasts from cougar.

**Primer sets**	**Species**	**Sequence**	**Amplicon size in bp**	**Annealing temperature in ^o^C**	**NCBI accesion number**
SDHA	*Felis silvestris*	F: 5’- GGACCATGAATTTGACGCGG-3’	103	59	XM_011287219.1
		R: 5’- TCGGAGCCTTTCACAGTGTC -3’			
OCT4	*Felis silvestris*	F: 5’- TGCAGCTCAGTTTCAAGAACA -3’	112	52	NM_001173441.1
		R: 5’- ACAAGTGTCTCTGCTTTGCATA -3’			
NANOG	*Felis silvestris*	F: 5’- ATGCACCCTTGCGAATGTCA -3’	120	55	NM_001173442.1
		R:5’- TTACTCTGGGGCTGGTGGAA -3’			
SOX2	*Canis lupus*	F:5’-AACGGCAGCTACAGCATGAT-3´	138	57	XM_005639752.3
		R: 5’-CGAGCTGGTCATGGAGTTGTA-3’			
KLF4	*Felis silvestris*	F: 5`-GTCCCATCAGGAGTCAGTGG-3’	207	59	NM_001173444.1
		R: 5’-GTCCAATTCAGGCCGAAGGA-3’			
CD44	*Felis silvestris*	F: 5´- TCGAGGCACCCCATTTCATAGACA -3´	128	60	XM_019812274.2
		R: 5´-ATCAGCTGGCTACTCTGTTGGACT-3´			
CD90	*Puma concolor*	F: 5´- AGCACGTGATCTTTGGCACTATGG -3´	134	59	XM_025929117.1
		R: 5-´ACATGTGTACATCCCCTCGTCCTT-3’			
CD105	*Puma concolor*	F: 5´- ATCACCTTTGGCGCCTTCCTTATC -3´	144	59	XM_025913844.1
		R: 5´-GTGGTTGGTGCTACTGCTTTCTGA-3’			
MHCII	*Puma concolor*	F: 5’-TGAGCTGAAGTGGAGATGCTGACA-3’	138	60	XM_025926082.1
		R: 5´-ACTGAACCCAGGGCAAACCAAA-3’			

### Immunocytochemistry

Immunohistochemistry was performed to detect OCT4 and SOX2 proteins. Cells were fixed with 4% paraformaldehyde for 5 min and incubation was carried out for 1 h with polyclonal primary antibodies, anti-OCT4 (1:200, Thermo Scientific, PA1-16943) and anti-SOX2 (1:25, Thermo Scientific, PA5-17282) followed by a 1h incubation with secondary anti-rabbit IgG-HRP conjugated (1:1000, Thermo Scientific, PA31463). Controls included: 1) no primary antibody, and incubation with the same secondary antibody and conditions as above and 2) specific primary antibodies (same conditions as above) and unspecific anti-mouse IgG (1:1000, Thermo Scientific PA 31430). The same primary antibodies had been tested previously in our laboratory (Cabezas et al., 2014). Nuclei and cytoplasm were counterstained with hematoxylin-eosin.

## Results

**Figure 1:**
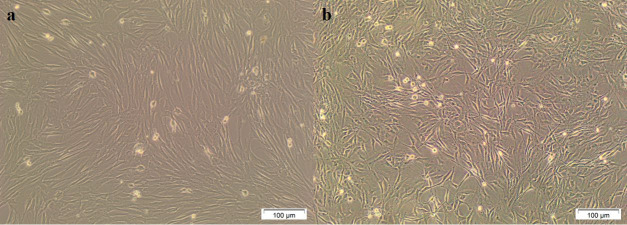
Cougar AMSC and fibroblasts cultured *in vitro*. (a) AMSC from cougar at P1; (b) Fibroblasts from cougar at P1.

AMSCs from cougar attached to the plastic surface of culture dishes and displayed fibroblastic morphology, with a spindle-like form, polygonal, and elongated shape. This morphology was also found in skin fibroblasts isolated from the same animal and remained cat fibroblasts and MSCs (Figure 1).

AMSCs grew fast after initial plating. After P5 cells showed signs of senescence and started to detach from the culture dish. The cell doubling time for fibroblasts was longer than for AMSC (Table 3).

**Table 3:** Cell doubling time for cougar fibroblasts and AMSC in hours. The cell doubling time between P0 and P1 for fibroblasts could not be calculated since the culture was established from skin explants and was defined as P0.

**Cell type/passage**	**P1**	**P2**	**P3**	**P4**	**P5**
AMSC	20.67	31.08	32.26	46.45	32
FIBROBLASTS		42.85	102.0	130.3	192

**Figure 2:**
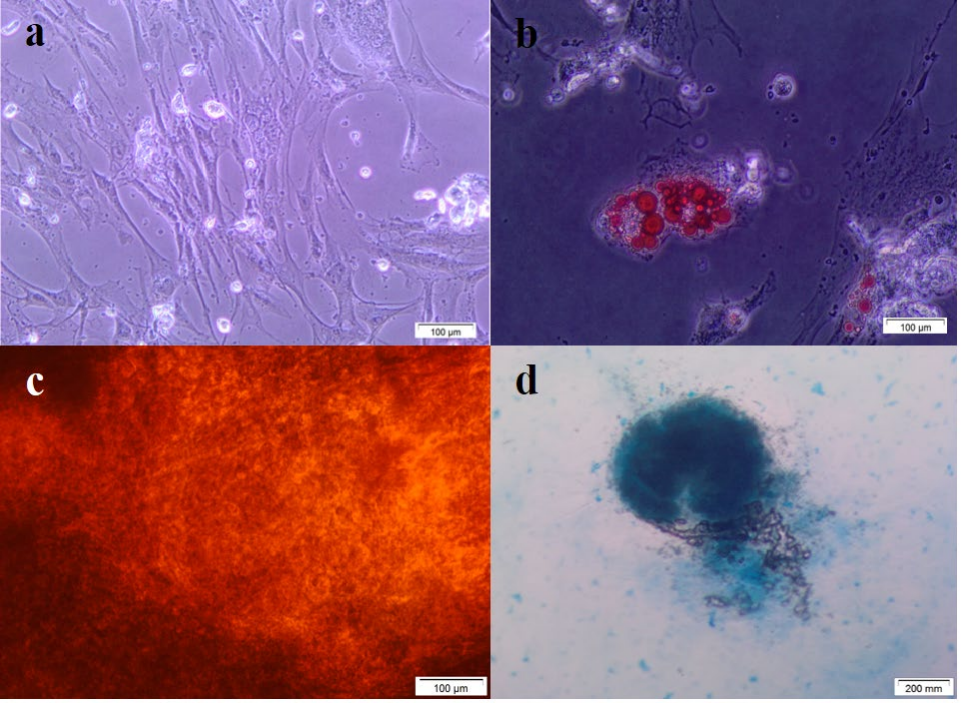
Multilineage differentiation of cougar AMSC. Cells showed differentiation capacity towards adipogenic, chondrogenic and osteogenic lineages. (a) Control without differentiation; (b) In adipogenic differentiation cells showed an increased granularity and larger intracellular oil droplets stained red by Oil Red; (c) Osteogenic induction was evidenced by calcium phosphate deposit stained red by Alizarin Red; (d) Aggregates with proteoglycan content after 28 days of chondrogenic induction culture showed intense Alcian Blue staining.

Cougar AMSCs displayed differentiation capacity towards adipogenic, osteogenic and chondrogenic lineages. Presence of lipid vacuoles was evident at day 7 of culture in the differentiation medium and further confirmed after Oil Red stain. Alizarin Red stained calcium deposits at day 21, whereas control cells remained without morphological changes and negative to staining. Pellet formation was identified in chondrogenic differentiation and glycosaminoglycans were stained by Alcian blue (Figure 2).

**Figure 3:**
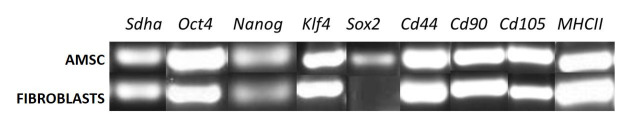
Agarose gel electrophoresis of the comparative expression of selected transcripts from AMSC and dermal fibroblasts from *Puma concolor* . Pluripotency genes: *Oct4, Nanog, Klf4 and Sox2.* Surface markers: *CD44, CD90 and* Major histocompatibility complex *MHCII* . Sdha was employed as a housekeeping. Molecular weight ladder is not shown, the length of the amplicons for each transcript is indicated in Table 2.

Pluripotent genes *Oct4, Nanog, Sox2* and *Klf4* and cell surface markers (*Cd44, Cd90, Cd105* and *MHCII)* were detected by RT-PCR in AMSC (Figure 3). *Oct4, Nanog and Klf4* mRNA were detected also in fibroblast cells from cougar, whereas *Sox2* was not. All of the surface markers analyzed (*Cd44*, *Cd90*, *Cd105* and *MHCII*) were detected also in fibroblasts (Figure 3).

**Figure 4:**
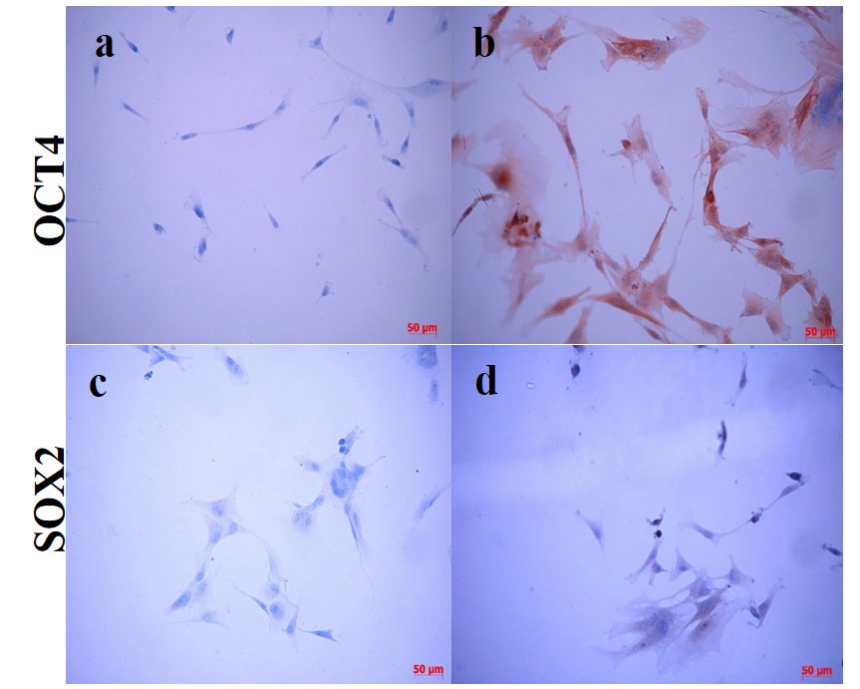
Detection of OCT4 and SOX2 by immunocytochemistry in AMSC from *Puma concolor*. (a and c) negative controls of expression for OCT4 and SOX2 respectively; the cells were fixed, primary antibody was omitted and the cells were incubated with the secondary anti-rabbit HRP sheep conjugated antibody and revealed with DAB; (b) Strong detection of OCT4 in cytoplasm and nuclei and (d) SOX2 immunodetection in the cytoplasm of some cells. Antibodies, catalogue numbers and specificity are described in materials and methods.

Regarding protein analysis, OCT4 was detected in AMSCs both in cytoplasmic and nuclear location, while SOX2 was mildly expressed only at cytoplasmic level (Figure 4).

## Discussion

Here we report for the first time partial characterization of AMSCs from cougar. The isolation of AMSCs from subcutaneous and abdominal tissue of domestic and wild cats (*Felis nigripes* and *Leopardus guigna*) was previously reported (Kono et al., 2014; Gómez et al., 2015; Echeverry et al., 2019). Here we isolated and characterized MSCs isolated from adipose tissue of *Puma concolor*. Cells showed similar morphology and growth kinetics to MSCs isolated from other felines like domestic cats and to fibroblasts. (Gómez et al., 2015; Clark et al., 2017; Lee et al., 2018; Echeverry et al., 2019). Noticeably, upon reaching confluence, starting at passages 4 and 5, growth was inhibited by contact and the cells detached from the plaque.

Our findings regarding expression of pluripotent and surface marker genes of cougar AMSCs are consistent with previous reports (Gómez et al., 2015; Lee et al., 2018; Echeverry et al., 2019). Others reported the expression of *Oct4, Nanog*, and *Klf4* in fibroblast cells in humans (Page et al., 2009; Ambady et al., 2010; Rodríguez-Álvarez et al., 2013), and bovine species (Rodríguez-Álvarez et al., 2013). Basal levels of *Nanog* mRNA expression have been previously reported in fibroblasts of domestic cat (Gómez et al., 2015; Echeverry et al., 2019). Therefore our findings concerning these markers are in line with common literature for AMSCs.

Interestingly *Sox2* mRNA expression were not reported earlier in AMSCs from wild cats. Conversely domestic cat, AMSC expressed *Sox2* in early passages (P1-P3) but expression decreased after continuous culture (>P5) (Lee et al., 2018). In the present study *Sox2* and pluripotent genes were evaluated at second passage only, therefor for these conditions, this agrees with the report of Lee et al., 2018, we did not attempt to identify *Sox2* in later passages.

In human and bovine fibroblasts, OCT4 protein expression was reported (Rodríguez-Álvarez et al., 2013). Here we detected OCT4 and SOX2 proteins in the cultured AMSC. OCT4 was localized both in cytoplasm and nucleus of the AMSCs, while SOX2 was found only in the cytoplasm. Previous studies had reported OCT4 protein in the cytoplasm of fibroblasts cells, one explanation for this finding is the presence of OCT4 pseudogenes in bovine cells (Yadav et al., 2005). In human *Oct4* encodes two different splice variants, described initially as *Oct4A* and *Oct4B*, where *Oct4B* is localized mainly in the cytoplasm of somatic cells (Lee et al., 2006). It has been reported in mouse embryonic stem cells that SOX2 can shift its localization pattern in early development from a cytoplasmic to a more nuclear distribution to contribute to pluripotency (Thevenet et al., 2004). Finding SOX2 at the cytoplasmic level may suggest response to differentiation signals or regulation of SOX2 via ubiquitination and proteasomal degradation (Baltus et al., 2009). For this reason, the finding of SOX2 in cougar AMSC cannot necessarily be attributed to multipotency features in these cells until further analysis is performed.

We found expression of *Klf4* in fibroblasts and AMSC, this is coincident with Gómez et al., 2015, who found this gene expressed in fibroblasts and AMSC from cats. Probably the expression of *Klf4* is not pivotal to differentiate AMSC from fibroblasts in the feline species.

Further, we found mRNA expression of *MHCII* in both AMSC and fibroblasts contradictorily to results reported by other authors (Clark et al., 2017). However, it was not possible to confirm the expression of this marker at the protein level. Lack of expression of MHCII in AMSCs may ensure a beneficial effect in allogeneic cell therapies due to their low immunogenicity (Rutigliano et al., 2013). Others surface markers as MHCI and CD45 at mRNA and protein level needs to be evaluated.

Not shown here, we attempted to detect, CD proteins CD44, CD90, MHCI and MHCII, by cell cytometer using antibodies raised against human said antigens, but they were not reliably detected. One possible explanation is the lack or low specificity of the used antibodies to feline antigenic epitopes (data no show). Specific species antibodies are required to perform more complete characterization of wild cat AMSC.

Cougar AMSCs showed multilineage differentiation potential towards adipogenic, osteogenic and chondrogenic lineage. The same was previously reported for domestic and wild cats (Kono et al., 2014; Gómez et al., 2015; Sato et al., 2016; Echeverry et al., 2019), but this is the first report of such differentiation for puma cells.

## Conclusion

This study describes for the first time characterization of some biological attributes of presumed cougar ASMCS including growth kinetics and morphology, expression of pluri- and multipotent markers and tri-lineage mesodermal differentiation potential. Our results indicate that adipose tissue of *Puma concolor* contains AMSCs, which can be isolated, expanded and differentiated and thus might be of value for regenerative therapies in this species.
